# Mr. Hamilton on Mercury in Obstinate Vomiting

**Published:** 1813-08

**Authors:** W. Hamilton

**Affiliations:** Surgeon. Ipswich


*To the Editors of the Medical and Physical Journal.*

GENTLEMEN,

ON looking over your last Number, (for July,) I find there a case of obstinate *vomiting* related by Dr. Yeats; in which, after having used many ineffectual remedies, he at length discovered the utility of mercury, in arresting the progress of so obstinate a disease. As a farther proof of the success of this practice, when properly directed,

I shall

I shall here add a few cases, which will at once convince any unprejudiced observer of its good effects in such cases.

Mrs. Chesen had for many years been affected with a stomach complaint, attended with vomiting. At length she became quite confined to bed, and regularly vomited every thing taken. She also complained of much pain at the pit of the stomach, with much oppression over the whole epigastric region. During the first week of her confinement, I tried all the common remedies used in such complaints, viz. purging, anodynes, cardiacs, and tonics, without the least benefit. As she appeared now in a hapless condition, I determined to lay all other medicines aside, and put her under the influence of mercury as soon as possible; by which I knew, from former experience, I could at least stop the progress of her disease for a time. She was therefore ordered two grains of calomel, with half a grain of opium, every three hours, in form of a small pill, which remained on the stomach, as no liquid was taken more than could possibly be avoided. In about forty-eight hours, the mouth began to be affected, and the vomiting to subside. The calomel was continued for another day, when it was laid aside, her mouth being now her chief complaint. No more vomiting appeared, and all the load at the stomach, as well as the pain, ceased. Her mouth remained sore for a week or ten days, during which she took some weak decoction of bark, and recovered her strength rapidly.

2. Mrs. Lanham had been ill of similar complaints for near a fortnight when I first visited her. She was now quite confined to bed, and unable to retain any thing upon her stomach. As she appeared almost reduced to a skeleton, I first attempted to check the vomiting by tonics and anodynes, as her bowels were pretty regular; I soon, however, found that these had no effect, and without farther delay gave her the calomel in doses of four grains with one of opium every four hours, with a view to affect the mouth as soon as possible. The case being severe, it required even at this rate almost a week's perseverance before the mouth was affected, when, as in the former instance, all her retchings and vomitings began to subside. She now omitted the calomel, and took the decoct.cinchon. as a tonic, which completely restored her in about a fortnight more.

N.B. In the first part of her complaint she vomited a stomach-worm of the teres kind, which seemed, however, to have little cause in producing her complaint, as the sickness and vomiting remained equally severe till the mouth became affected.

3. Mrs. Allen.—This was a still more obstinate case, as it was was more chronic. She had been for months attended by a physician and surgeon, without any permanent benefit. She was confined to bed, and appeared in the utmost danger. I was called up to her in the night, and, as she appeared dying, the retching and vomiting having continued incessant for several hours, with a burning heat at her stomach, I at first attempted to allay it by giving several drams of tinct. opii, which was no sooner taken than it was ejected. In this state, knowing not what to recommend, I gave her cold water only, in small quantities, as fast as she could swallow it, and applied cloths wetted with vinegar over the region of the stomach, which were renewed every ten minutes. This produced such a shock as to check the vomiting for several hours, when I seized the opportunity of introducing the calomel and opium, as in the last case. The desired effect was produced in about a week, and the result was equally pleasing. Here, as in the former instances, just in proportion as the mouth became affected, in the same proportion did all her other complaints subside. She afterwards took the bark, and has had no return of the disease since, although now more than five months.

4. Mrs. Bond had been attended by a medical gentleman for near a fortnight, who finding no abatement of the disease, ingenuously confessed his doubts with regard to her recovery. Under these circumstances I was called in all haste, when I found her complaining of much pain over the epigastric region, a sensation of weight or oppression at the pit of the stomach, constant retching and vomiting on taking any thing into it, pulse rapid, skin hot, bowels regular. On pressing the hand over the region of the stomach and umbilicus, I could easily perceive a considerable enlargement of the left ovarium and spleen, which latter seemed to press against the stomach, and was no doubt the chief cause of vomiting in this case, especially as the sickness was only induced by taking something into the stomach which naturally enlarged it. Here, then, I had two strong inducements to use the calomel: first, to prevent organic disease; and, secondly, to arrest the vomiting, which was in this instance a necessary consequence of the former. She was therefore ordered to lay aside all medicines except the calomel, which she took at the rate of twenty grains a-day, with three of solid opium, in form of three pills, at the distance of six hours for six days, without either materially affecting her mouth, or making much impression on the disease. The sickness, however, was considerably abated, and the oppression at the stomach nearly removed. Here the calomel was omitted for two days, fearing a violent ptyalism would ensue on its

 farther farther perseverance. The day after its omission, the vomiting and oppression seemed to return, when I immediately determined on its resumption, as I knew I had gained no hing till the mouth was affected. She now took ten grains with three of opium every twenty-four hours, during five more days, when the mouth at length appeared affected, and it was again omitted. From this period the mouth continued sore for near a fortnight, during which she drank some decoct. cinchon. and recovered rapidly, without ever mentioning vomiting or load at the stomach from the commencement of the ptyalism. In this case, which was truly obstinate, the only difficulty was to induce the salivation, she having taken upwards of 180 grains of calomel before the violence of the disease allowed it to affect the mouth; nor was it carried off by the bowels, as they were generally costive during the fortnight of its exhibition; it acted only as a gentle sudorific. The subjects of these cases, although they have all occurred within the last six months, now enjoy the most perfect health. And in every instance of this practice, the convalescence seems particularly short when aided with the bark.

Although I have been for some time making experiments and observations on the effects of this medicine, which promise the happiest result, I shall here conclude, without entering at present into its *modus operandi*, by stating one fact which is as invariable as the law of nature on which it is founded, viz. in proportion as a ptyalism is induced, in the same proportion will the original disease subside.

*Ipswich*, Your's respectfully,

*July* 6, 1813. W. HAMILTON, Surgeon.

